# Supplementary material for: Staple Food Preference and Obesity Phenotypes: The Regional Ethnic Cohort Study in Northwest China
Source: Nutrients. 2022 Dec 9;14(24):5243. doi: 10.3390/nu14245243 (PMC9784345; doi:10.3390/nu14245243)
Supplement: Supplementary file 1 [file nutrients-14-05243-s001.zip › SupplementaryFile.docx]

Supplementary Materials

**Staple food preference and obesity phenotypes: the Regional Ethnic Cohort Study in Northwest China**

Kun Xu^a^, Binyan Zhang^a^, Yezhou Liu^a^, Baibing Mi^a^, Yutong Wang^a^, Yuefan Shen^a^, Guoshuai Shi^a^, Shaonong Dang^a^, Xin Liu^a^†, and Hong Yan^a, b^†

a. Key Laboratory for Disease Prevention and Control and Health Promotion of Shaanxi Province, Department of Epidemiology and Biostatistics, School of Public Health, Global Health Institute, Xi’an Jiaotong University Health Science Center, 76 West Yanta Road, 710061, Xi’an, Shaanxi, China.

b. Nutrition and Food Safety Engineering Research Center of Shaanxi Province, Key Laboratory of Environment and Genes Related to Diseases, Xi’an Jiaotong University, 76 West Yanta Road, 710061, Xi’an, Shaanxi, China.

†**Address correspondence to: Prof. Xin Liu**, PhD, Department of Epidemiology and Biostatistics, School of Public Health, Xi’an Jiaotong University Health Science Center, 76 West Yanta Road, Xi’an, Shaanxi 710061, P. R. China. Phone: +86-29-82655108; Fax: +86-29-82655101. Email: [xinliu@xjtu.edu.cn](mailto:xinliu@xjtu.edu.cn); or **Prof Hong Yan,** PhD, Department of Epidemiology and Biostatistics, School of Public Health, Xi’an Jiaotong University Health Science Center, 76 West Yanta Road, 710061, Xi’an, Shaanxi, China. Phone: +86-29-8265-5001; fax： +86-29-82655104 Email: [xjtu_yh.paper@aliyun.com](mailto:xjtu_yh.paper@aliyun.com).

**Supplemental Tables**

## Table S1. The associations of staple food preference with the risks of excessive body fat in men by provinces.

| Province | Models | Types of staple food | | | Trend *P* |
| --- | --- | --- | --- | --- | --- |
|  |  | wheat | both | rice |  |
| Shaanxi | Cases (n (%)) | 2011 (30.35%) | 1281 (18.49%) | 416 (16.02%) |  |
|  | OR (95%CI)^1^ | 1.000 | 0.996 (0.908, 1.091) | 0.658 (0.579, 0.747) | <0.001 |
|  | OR (95%CI)^2^ | 1.000 | 0.999 (0.910, 1.096) | 0.693 (0.607, 0.791) | <0.001 |
|  | OR (95%CI)^3^ | 1.000 | 0.999 (0.910, 1.096) | 0.692 (0.606, 0.790) | <0.001 |
| Ningxia | Cases (n (%)) | 543 (50.56%) | 2914 (64.02%) | 22 (78.57%) |  |
|  | OR (95%CI)^1^ | 1.000 | 1.617 (1.411, 1.853) | 3.809 (1.529, 9.492) | <0.001 |
|  | OR (95%CI)^2^ | 1.000 | 1.565 (1.364, 1.795) | 3.657 (1.462, 9.146) | <0.001 |
|  | OR (95%CI)^3^ | 1.000 | 1.608 (1.400, 1.846) | 3.744 (1.495, 9.376) | <0.001 |
| Gansu | Cases (n (%)) | 2432 (48.04%) | 1464 (45.16%) | 109 (46.98%) |  |
|  | OR (95%CI)^1^ | 1.000 | 0.892 (0.816, 0.975) | 0.963 (0.738, 1.255) | 0.028 |
|  | OR (95%CI)^2^ | 1.000 | 0.807 (0.735, 0.887) | 0.836 (0.637, 1.096) | <0.001 |
|  | OR (95%CI)^3^ | 1.000 | 0.799 (0.727, 0.879) | 0.824 (0.628, 1.081) | <0.001 |
| Xinjiang | Cases (n (%)) | 3487 (44.90%) | 769 (24.73%) | 77 (18.42%) |  |
|  | OR (95%CI)^1^ | 1.000 | 0.946 (0.837, 1.069) | 1.402 (0.962, 2.044) | 0.920 |
|  | OR (95%CI)^2^ | 1.000 | 0.898 (0.793, 1.016) | 1.287 (0.880, 1.882) | 0.435 |
|  | OR (95%CI)^3^ | 1.000 | 0.896 (0.792, 1.015) | 1.284 (0.878, 1.878) | 0.422 |
| Qinghai | Cases (n (%)) | 360 (58.44%) | 117 (41.64%) | 24 (36.92%) |  |
|  | OR (95%CI)^1^ | 1.000 | 0.741 (0.527, 1.042) | 0.953 (0.472, 1.925) | 0.216 |
|  | OR (95%CI)^2^ | 1.000 | 0.727 (0.512, 1.034) | 0.949 (0.463, 1.945) | 0.212 |
|  | OR (95%CI)^3^ | 1.000 | 0.759 (0.532, 1.083) | 0.904 (0.440, 1.857) | 0.241 |

^1^**Model 1**, adjusted for age, ethnic; ^2^**model 2**, additionally adjusted for drinking status, smoking status, family income, and education level; ^3^**model 3**, further adjusted for physical activity (MET-hr /day).

## Table S2. The associations of staple food preference with the risks of central obesity in women by provinces.

| Province | Models | Types of staple food | | | Trend *P* |
| --- | --- | --- | --- | --- | --- |
|  |  | wheat | both | rice |  |
| Shaanxi | Cases (n (%)) | 6853 (55.88%) | 3391 (36.71%) | 1390 (35.35%) |  |
|  | OR (95%CI)^1^ | 1.000 | 0.862 (0.810, 0.918) | 0.795 (0.730, 0.865) | <0.001 |
|  | OR (95%CI)^2^ | 1.000 | 0.918 (0.862, 0.979) | 0.828 (0.758, 0.904) | <0.001 |
|  | OR (95%CI)^3^ | 1.000 | 0.921 (0.864, 0.982) | 0.835 (0.765, 0.912) | <0.001 |
| Ningxia | Cases (n (%)) | 1059 (71.99%) | 5074 (73.46%) | 55 (75.34%) |  |
|  | OR (95%CI)^1^ | 1.000 | 1.095 (0.965, 1.243) | 1.149 (0.666, 1.982) | 0.156 |
|  | OR (95%CI)^2^ | 1.000 | 1.102 (0.970, 1.252) | 1.155 (0.669, 1.994) | 0.134 |
|  | OR (95%CI)^3^ | 1.000 | 1.103 (0.971, 1.253) | 1.165 (0.675, 2.011) | 0.128 |
| Gansu | Cases (n (%)) | 3665 (54.18%) | 2234 (52.86%) | 133 (52.78%) |  |
|  | OR (95%CI)^1^ | 1.000 | 0.965 (0.892, 1.043) | 1.064 (0.825, 1.372) | 0.589 |
|  | OR (95%CI)^2^ | 1.000 | 1.090 (0.999, 1.188) | 1.265 (0.975, 1.642) | 0.018 |
|  | OR (95%CI)^3^ | 1.000 | 1.077 (0.987, 1.175) | 1.250 (0.963, 1.623) | 0.036 |
| Xinjiang | Cases (n (%)) | 8670 (80.66%) | 3754 (77.90%) | 638 (76.41%) |  |
|  | OR (95%CI)^1^ | 1.000 | 0.933 (0.842, 1.034) | 0.903 (0.747, 1.092) | 0.152 |
|  | OR (95%CI)^2^ | 1.000 | 0.959 (0.864, 1.064) | 0.925 (0.764, 1.120) | 0.334 |
|  | OR (95%CI)^3^ | 1.000 | 0.957 (0.862, 1.062) | 0.923 (0.762, 1.118) | 0.320 |
| Qinghai | Cases (n (%)) | 549 (53.56%) | 279 (51.01%) | 69 (48.59%) |  |
|  | OR (95%CI)^1^ | 1.000 | 1.041 (0.837, 1.295) | 1.042 (0.718, 1.511) | 0.722 |
|  | OR (95%CI)^2^ | 1.000 | 1.094 (0.874, 1.369) | 1.194 (0.814, 1.752) | 0.283 |
|  | OR (95%CI)^3^ | 1.000 | 1.091 (0.871, 1.366) | 1.197 (0.816, 1.758) | 0.284 |

^1^**Model 1**, adjusted for age, ethnic; ^2^**model 2**, additionally adjusted for drinking status, smoking status, family income, and education level; ^3^**model 3**, further adjusted for physical activity (MET-hr /day).

**Supplemental Figures**

**
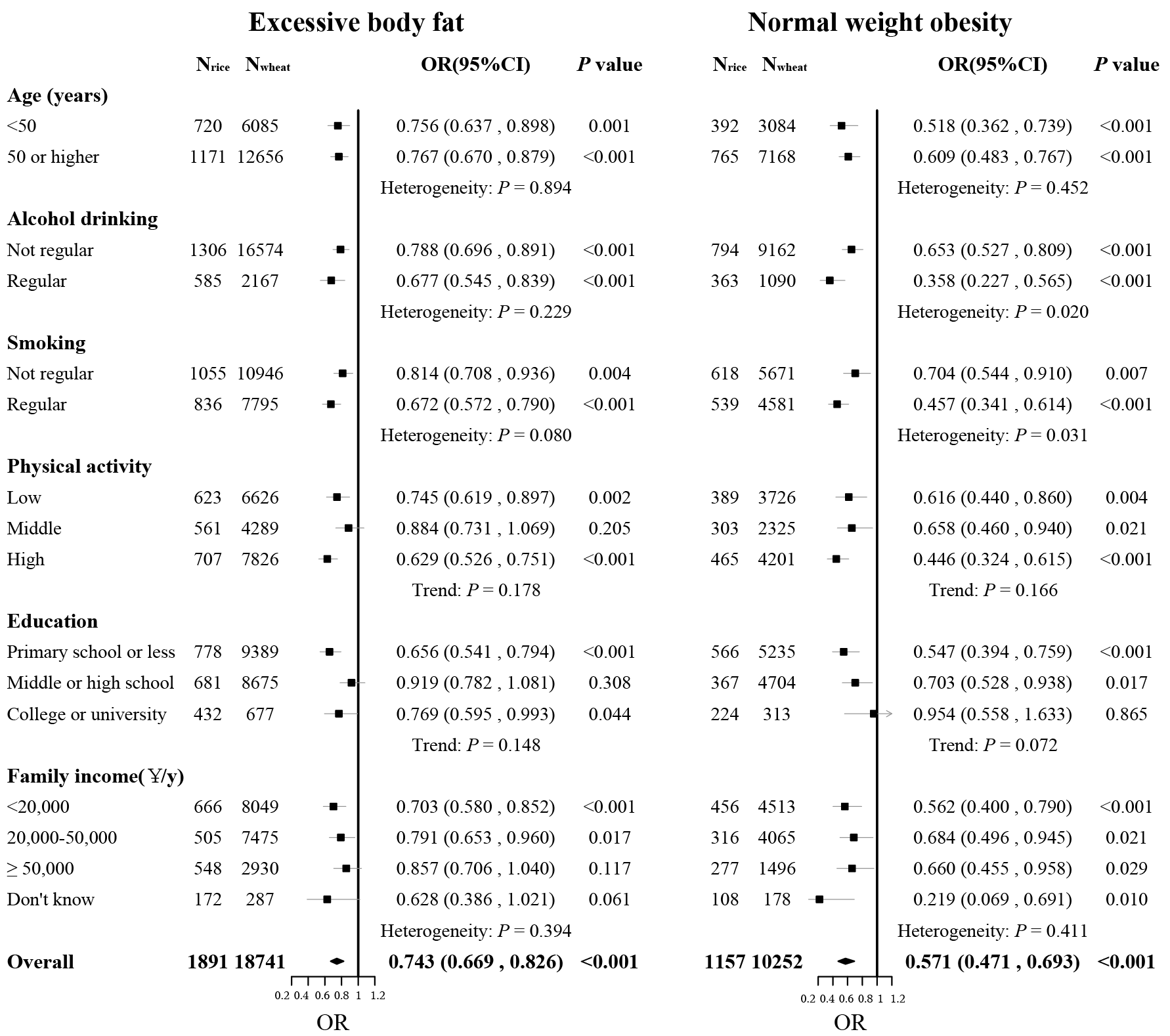
**

**Figure S1. Adjusted odds ratios (ORs) of excessive body fat and normal weight obesity in men for rice preference *vs.* wheat preference stratified by population characteristics.** Models were adjusted for age, ethnic, province, drinking status, smoking status, family income, education level, and physical activity (MET-hr /day).


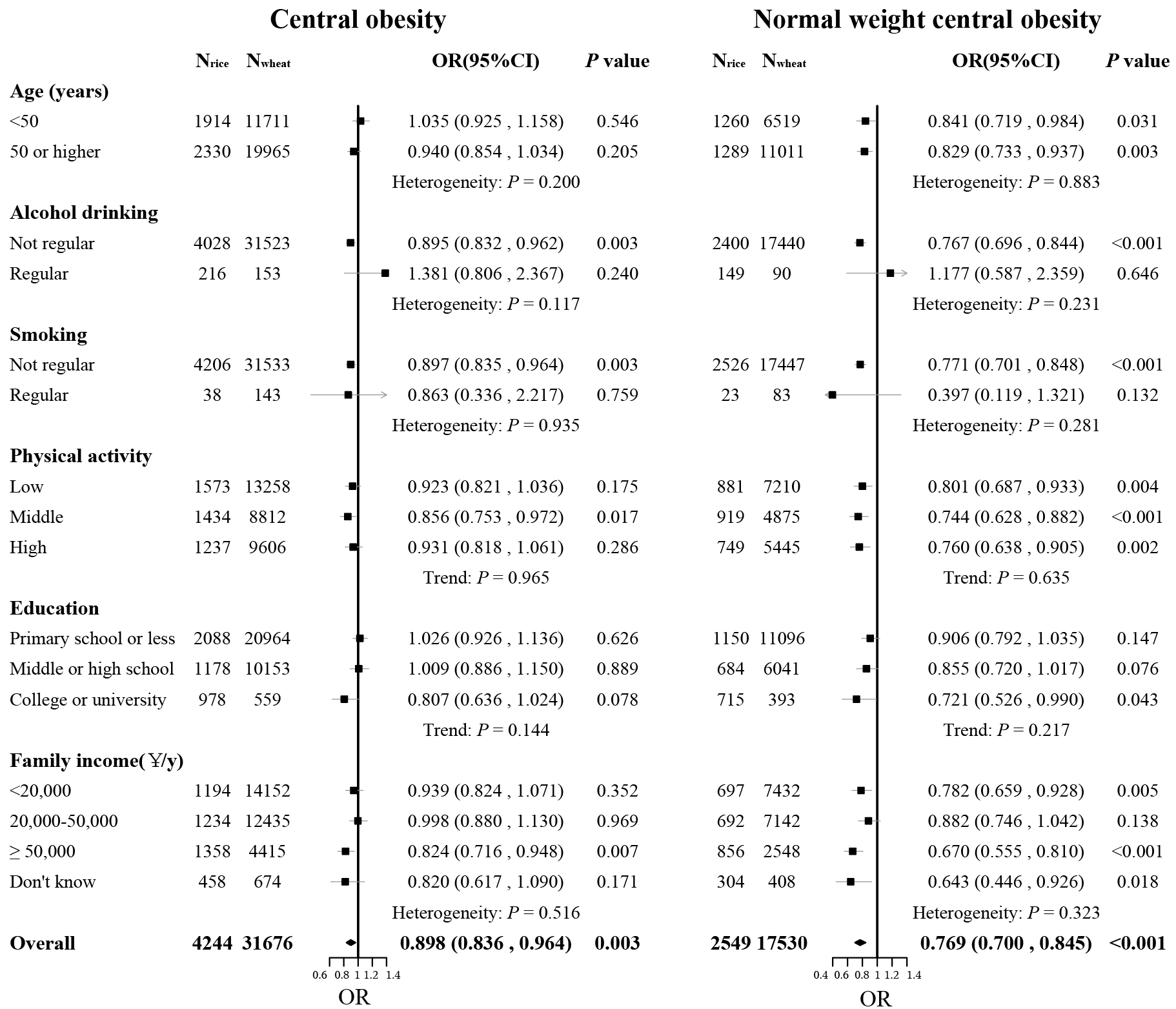


**Figure S2. Adjusted odds ratios (ORs) of central obesity and normal weight central obesity in women for rice preference *vs.* wheat preference stratified by population characteristics.** Models were adjusted for age, ethnic, province, drinking status, smoking status, family income, education level, physical activity (MET-hr /day).

**
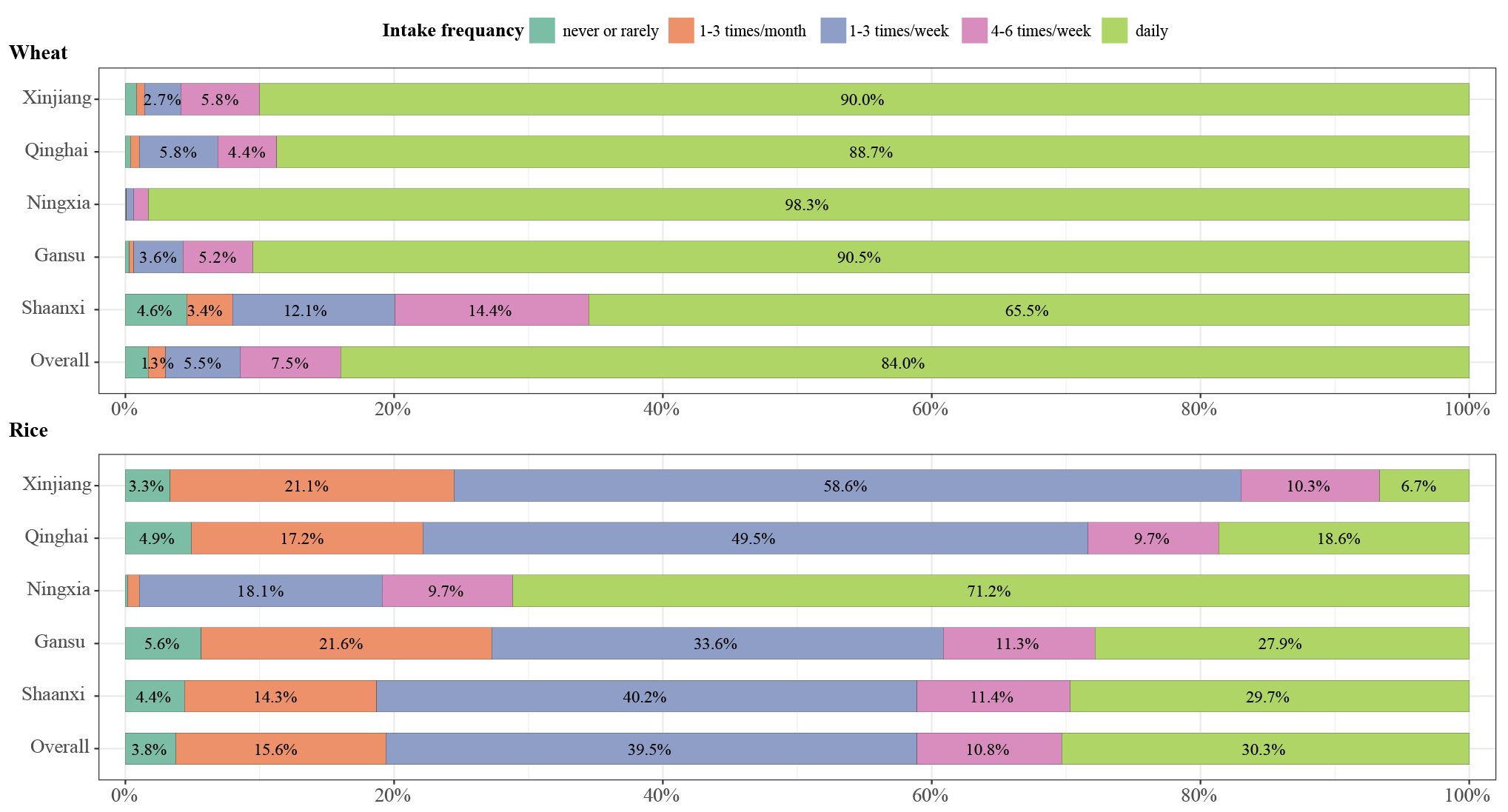
**

**Figure S3. The intake frequency of rice and wheat totally and by provinces.**

**
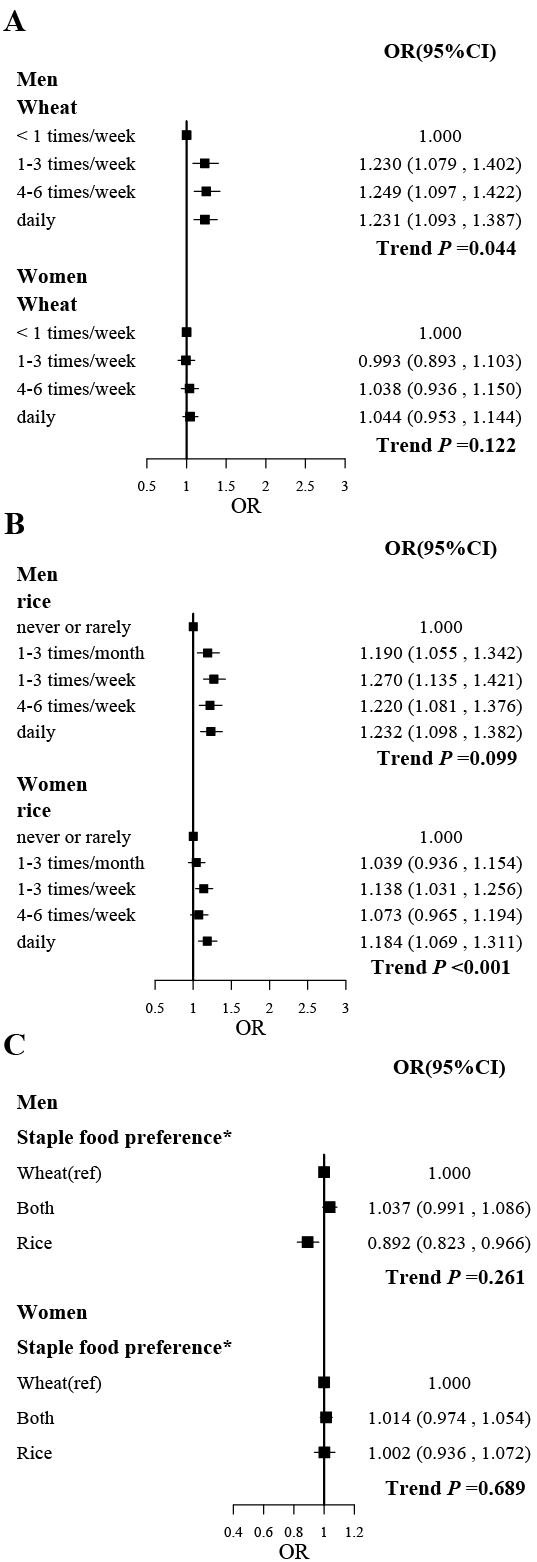
**

**Figure S4. ORs (95%CIs) of overweight/obesity (BMI > 25kg/m^2^) for wheat intake (A), rice intake (B), and staple food preference (C).** *Staple food preference：rice, consuming rice every day or 4-6 times per week and consuming wheat less than 4-6 times per week; wheat, consuming wheat every day or 4-6 times per week and consuming rice less than 4-6 times per week; both, the rest of the participants, which means they consumed rice and wheat with similar frequency. Models were adjusted for age, province, ethnic, drinking status, smoking status, family income, and education level and physical activity (MET-hr /day).
